# Manual therapy as a conservative treatment for adolescent idiopathic scoliosis: a systematic review

**DOI:** 10.1186/1748-7161-3-2

**Published:** 2008-01-22

**Authors:** Michele Romano, Stefano Negrini

**Affiliations:** 1 ISICO (Italian Scientific Spine Institute), Milan, Italy

## Abstract

**Background:**

The treatment of adolescent idiopathic scoliosis is contingent upon many variables. Simple observation is enough for less serious curvatures, but for very serious cases surgical intervention could be proposed. Between these there is a wide range of different treatments. Manual therapy is commonly used: the aim of this paper is to verify the data existing in the literature on the efficacy of this approach.

**Methods:**

A systematic review of the scientific literature published internationally has been performed. We have included in the term manual therapy all the manipulative and generally passive techniques performed by an external operator. In a more specific meaning, osteopathic, chiropractic and massage techniques have been considered as manipulative therapeutic methods. We performed our systematic research in Medline, Embase, Cinhal, Cochrane Library, Pedro with the following terms: idiopathic scoliosis combined with chiropractic; manipulation; mobilization; manual therapy; massage; osteopathy; and therapeutic manipulation. The criteria for inclusion were as follows: Any kind of research; diagnosis of adolescent idiopathic scoliosis; patients treated exclusively by one of the procedures established as a standard for this review (chiropractic manipulation, osteopathic techniques, massage); and outcome in Cobb degrees.

**Results:**

We founded 145 texts, but only three papers were relevant to our study. However, no one of the three satisfied all the required inclusion criteria because they were characterized by a combination of manual techniques and other therapeutic approaches.

**Conclusion:**

The lack of any kind of serious scientific data does not allow us to draw any conclusion on the efficacy of manual therapy as an efficacious technique for the treatment of Adolescent idiopathic scoliosis.

## Background

Idiopathic scoliosis is a pathology that affects approximately 2% of adolescents [1,2]. Various treatment procedures are proposed, according to severity of curvature, hump height, age at diagnosis, spinal stiffness and skeletal maturity. One of the major problems in the treatment of this pathology is the lack of a reliable prognostic tool for its possible evolution. There are scolioses that, once discovered, don't worsen anymore, whereas others are characterized by a rapid and dramatic worsening – especially during the pubertal growth spurt – or that continue to worsen in adulthood. This particular variety of behaviours allows us to employ two alternative methods of therapeutic approach:

1. The patients are not subjected to screening or to previous treatments. This forces the persons who are affected by serious scoliosis to begin treatment later, causing negative repercussions on the final results or the need to perform an important surgical intervention.

2. The patients are subjected to a screening (school, family, pediatrician); all risky scolioses are identified (namely those that exceed a certain degree according to age) and are referred to a program of conservative treatment, according to the potential risk of evolution. Furthermore, this procedure has a disadvantage in that it involves a long and expensive treatment even for the subject who is not affected by an progressive scoliosis.

The steps of non-surgical intervention can start with a simple observation, if no particularly important curvature is discovered and the subject is not at a particularly risky age. Alternatively, it is possible to adopt different therapeutic solutions, such as the use of braces [3-13], which are intended to correct spinal development when curvature evolution is not controlled through other techniques. Many studies have been conducted regarding the efficacy of such a therapeutic approach, whose validity is nevertheless confirmed by official medicine [5,8,14-16]. Another common therapeutic approach is the use of physical exercises [17-27] whose efficacy is not universally recognized, even if two systematic review [28] has been published during recent years. The results of these works do not allow us to state with reasonable certainty that this procedure will represent an efficacious treatment for idiopathic scoliosis, given that the studies on which the aforementioned review were based had been performed using different techniques. However, the possibility of comparing the best results from the various treated groups with the respective control groups encourages us to carry out new research with a better scientific design [28,29]. Besides the above-described treatments, it is possible to mention a range of alternative therapeutic proposals, such as the implantation of a dental bite or of podalic insoles. Their efficacy, however, has not been supported by published studies. A therapeutic approach that seems more and more common for the conservative treatment of adolescent idiopathic scoliosis includes manual techniques proposed by chiropractors and osteopaths. These techniques are favoured by their progressive popularity within the wider and wider panorama of alternative therapies [30-38].

The purpose of this study is to present a systematic review of the international scientific literature in order to verify the therapeutic efficacy of these procedures, which include all the manipulative and generally passive techniques performed by an external operator to treat idiopathic scoliosis. In a more specific meaning, osteopathic, chiropractic and massage techniques have been considered manipulative therapeutic methods.

## Methods

We conducted systematic research in the following databases: *Medline, Embase, Cinhal, Cochrane Library *and *Pedro*. The research was carried out in May of 2005 without time, meaning without fixing a starting date or imposing language restrictions. The key phrases were as follows: *Idiopathic scoliosis *(combined with *chiropractic*)*; manipulation; mobilization; manual therapy; massage; osteopathy; and therapeutic manipulation*. The research was completed through the analysis of the other papers published and available, thanks to the following specialized databases: *Index to Chiropractic Literature, Osteomed, Osteopathic Research Web*, and *N.C.C.A.M*.

The criteria for inclusion were as follows:

• Subjects: Diagnosis of Adolescent idiopathic scoliosis;

• Type of treatment received: Exclusively one of the procedures fixed as a standard for this review (chiropractic manipulation, osteopathic techniques, massage);

• Outcome: Cobb degrees;

• Type of study: Any kind of research.

The decision not to apply highly restrictive selection criteria but to include studies conducted with different procedures was determined through the presumption that homogeneous trials are seldom performed.

## Results

### Main results

A total of seventy-three papers were found in this research in PubMed/Medline (Table 1). In the other databases (Pedro, Cinhal, Cochrane Library, Embase), excluding papers already found in PubMed, we retrieved:

**Table 1:** Results of the research in PubMed according to the keywords used.

**Combination of keywords**	**Papers found**
*Idiopathic scoliosis *AND *chiropractic*	13
*Idiopathic scoliosis *AND *manipulation*	20
*Idiopathic scoliosis *AND *massage*	18
*Idiopathic scoliosis *AND *mobilization*	28
*Idiopathic scoliosis *AND *manual therapy*	3
*Idiopathic scoliosis *AND *therapeutic manipulation*	2

**Total of papers retrieved (excluding duplicates)**	**73**

• No reviews in *Cochrane Library;*

• Four papers in *Pedro;*

• Twenty papers in *Embase;*

• Fifty-three papers in *Index to Chiropractic Literature;*

• No papers in *Osteomed *and *Osteopathic Research Web*. In these two databases it was possible to find papers concerning adult scoliosis. In any case there were no research trials but only case reports or more general subjects.

• No papers in N.C.C.A.M. (National Centre for Complementary and Alternative Medicine).

After excluding duplicates, among the 145 texts we found, only three papers were relevant to our study. No one, however, satisfied all the required inclusion criteria. Nevertheless, we present these papers for possible future research.

### Possibly relevant papers not satisfying the inclusion criteria

Out of the three possibly relevant papers, two were non controlled studies (Table 2).

**Table 2:** Methodological characteristics and results of the two non controlled studies retrieved.

**Paper**	**Study design**	**No. subjects**	**Avg. age**	**Initial ° Cobb**	**Final ° Cobb**
Morningstar [31]	Non-controlled	19	15–65	28 average	average 11
Lantz [32]	Non-controlled	42	6–17	6–25	Improvement 0.9°

The first paper [31] is a non-controlled cohort study examining the combined effect of spinal manipulation and other procedures, such as traction and exercises, whose results don't give clear information about the efficacy of the specific manual therapy. The nineteen examined subjects (ranging in age from fifteen to sixty-five years) underwent three weekly treatment sessions for four to six weeks. The average Cobb angle at the beginning of treatment was 28°, and at the end it was 11° with an average improvement of 17°. The best improvement observed was 38°, and the smallest was 8°.

The second study [32] concerned a treatment combining chiropractic manipulation with podalic insoles, the latter being prescribed when curvature convexity concerned the shorter leg. All subjects received postural advice. The selected subjects numbered forty-two (twenty-six females, sixteen males) ranging in age from six to seventeen years, with curvatures between Cobb 4° and 22°. Treatment was executed for an average of fourteen months, intended as periods between the first session and the first follow-up, with an average of three sessions per month. The results showed an average improvement of 0.9°. Specifically, an improvement was observed in ten cases (19%), a worsening in six cases (11%) and no changes in thirty-seven cases (70%). Two cases showed an inversion of scoliosis on the frontal plane, with a transfer of the curvature to the opposite direction. These cases were considered as having improvement. On the basis of results, whose analysis was conducted by a single examiner, the authors stated that chiropractic manipulation, associated with the use of insoles and postural advice, did not produce an improvement in scoliotic curvature.

The third paper is a pilot study to explore patient recruitment and compliance, treatment standardization and outcome measure selection. Author's conclusion showed the viability for a larger randomized trial [39].

### Case reports

Moreover, we found three case reports [33,34,40] concerning the treatment of young subjects with idiopathic scoliosis: The former [34] showed the effect of a manipulative cycle during a period of seventeen months in association with an exercise program, and involved two adolescents having two extremely different types of scoliosis (16° and 60°). Among the many weak points of this study (only two subjects with very different scolioses and the use of combined treatment techniques), we can also mention that, as outcome instruments, the operators did not use objective methods as Cobb degrees but instead used visual evaluation and palpation, including shoulder asymmetry, axillae asymmetry and position of scapula angle. The authors observed that scoliosis decompensation was successfully stopped and that the effects of correction remained for 10 years.

The second case report [33] studies the effect of a manipulative cycle during a period of three months in association with a treatment involving night-time electric stimulations over a period of nine months on a patient with juvenile idiopathic scoliosis. The curvature progressed at a rate of 1.0 degree per month over the previous nine months. The patient's curvature was successfully stopped at 27 degrees and subsequently reversed to 17 degrees in the first three months of therapy.

Tha last paper [40] is a case series of 3 atypical patient: a left thoracic, idiopathic scoliosis after Harrington rod instrumentation, a left thoracic scoliosis secondary to Scheuermann's Kyphosis and a primary idiopathic thoracic levoscoliosis. Patients were treated with an active rehabilitation program including spinal manipulation and an external head and body weighting system. After 12 weeks of treatment authors found an apparent reduction in Cobb degrees in all 3 cases, but they admit the possibility of a placebo effect because of the lack of a control group.

## Discussion

None of the papers retrieved had the characteristics required for inclusion in our review. Indeed, we found only two research trials. Neither of the two had a control group, but each was characterized by a combination of manipulative procedures and other therapeutic approaches. The results of these two research studies are discordant. In the paper written by Morningstar et al [31]the result seems extremely positive (an average improvement of 17° Cobb after four to six weeks of treatment), while the conclusions of the study conducted by Lantz et al. [32] show an absolute ineffectiveness of the applied treatment. It must be stated that the first study had certain flaws: The recruited group of subjects presented an excessively wide age range, but no control group was designed and no follow-up observation was performed after treatment. Some doubts also arise as to the results, which seem so extraordinary to suggest a deep reflection to all health workers engaged in the conservative treatment of scoliosis throughout the world.

Case reports are not a valuable aid. Even when summing up single results, in the absence of homogeneous studies the data is absolutely inadequate (only two cases related to young patients, always characterized by the association of manual treatment with other techniques). None of the other papers allowed us to obtain supplementary information that could be deemed useful.

Generally, for manual therapy we can underline that even if we were to extend our inclusion criteria, the available papers are completely inadequate for a review. We can compare these results with those obtained using different conservative techniques, such as physical therapy or braces. Regarding physical exercises, few trials have been conducted as to their efficacy. We can mention just two systematic reviews [28,41]. The quality of the retrieved papers is not very high, given the lack of randomized controlled studies. However, the results are very interesting for future research and suggest the possible usefulness of exercises. The most recent review [41] published in 2005, asserts that at this state of the art, physical therapy and the application of a brace have the same reliability value. Of course, in the scientific literature we can find a greater number of papers on treatment using a brace. However, for this technique we still lack the certainty that the brace is an absolutely reliable therapeutic technique.

In conclusion, the data reported above does not allow us to evaluate the efficacy of manual therapy techniques in adolescent idiopathic scoliosis. These results confirm the urgent needof scientific studies with at least one control group, to be performes by operators using such techniques, to demonstrate their validity.

## Authors' contributions

Both authors participated in all phases of the study, excluding data collection, that was performed by MR alone.
